# Toward Biorecycling: Isolation of a Soil Bacterium That Grows on a Polyurethane Oligomer and Monomer

**DOI:** 10.3389/fmicb.2020.00404

**Published:** 2020-03-27

**Authors:** María José Cárdenas Espinosa, Andrea Colina Blanco, Tabea Schmidgall, Anna Katharina Atanasoff-Kardjalieff, Uwe Kappelmeyer, Dirk Tischler, Dietmar H. Pieper, Hermann J. Heipieper, Christian Eberlein

**Affiliations:** ^1^Department of Environmental Biotechnology, Helmholtz Centre for Environmental Research – UFZ, Leipzig, Germany; ^2^Interdisciplinary Ecological Center, TU Bergakademie Freiberg, Freiberg, Germany; ^3^Microbial Interactions and Processes Research Group, Helmholtz Centre for Infection Research – HZI, Braunschweig, Germany

**Keywords:** plastic, biorecycling, *Pseudomonas*, polyurethane, diaminotoluene, aromatics degradation, aromatic diamines

## Abstract

The fate of plastic waste and a sustainable use of synthetic polymers is one of the major challenges of the twenty first century. Waste valorization strategies can contribute to the solution of this problem. Besides chemical recycling, biological degradation could be a promising tool. Among the high diversity of synthetic polymers, polyurethanes are widely used as foams and insulation materials. In order to examine bacterial biodegradability of polyurethanes, a soil bacterium was isolated from a site rich in brittle plastic waste. The strain, identified as *Pseudomonas* sp. by 16S rRNA gene sequencing and membrane fatty acid profile, was able to grow on a PU-diol solution, a polyurethane oligomer, as the sole source of carbon and energy. In addition, the strain was able to use 2,4-diaminotoluene, a common precursor and putative degradation intermediate of polyurethanes, respectively, as sole source of energy, carbon, and nitrogen. Whole genome sequencing of the strain revealed the presence of numerus catabolic genes for aromatic compounds. Growth on potential intermediates of 2,4-diaminotoluene degradation, other aromatic growth substrates and a comparison with a protein data base of oxygenases present in the genome, led to the proposal of a degradation pathway.

## Introduction

Plastics are heavily used in our modern society and the global production rates increase since decades. With about 3.5 million tons polyurethanes were the fifth most demanded synthetic polymers in Europe in 2015 (Plasticseurope, 2016). The uses of polyurethanes are manifold with the major field of application being insulation materials. Common precursors used to synthesize polyurethanes are polyisocyanates and polyols together with additives such as catalysts, cross linkers and chain extenders, among others. Despite forming urethane bonds with the polyisocyanates, polyols additionally can contain ether or ester bonds, resulting in polyether or polyester polyurethanes, respectively. On the other hand, the polyisocyanate compounds can have an aliphatic, polycyclic or aromatic nature. Two of the most widely used diisocyanates for PU synthesis are 4,4′-methylene diphenyl diisocyanate (MDI) and toluene-2,4-diisocyanate (TDI) and their precursors 4,4′-diaminodiphenylmethane (MDA) and 2,4-diaminotoluene (2,4-TDA), respectively. Next to an alcohol and carbon dioxide, primary amines are also formed after chemical hydrolysis of the urethane bond (Marchant et al., 1987).

Post-consumer plastics are already a major challenge for the environment and will be an even bigger one in the future. The biodegradation is often hampered by the durability, crystallinity and macroscopic structure of the polymers. For polyurethanes, the diverse chemical composition increases the obstacles for both, biological and chemical recycling. Reports on the degradation of polyurethanes mostly focus on polyester-based ones, fungal as well bacterial and enzymatic hydrolysis were reported (Wang et al., 1997; Russell et al., 2011; Krasowska et al., 2012; Shah et al., 2013; Magnin et al., 2019). The biodegradation of polyether-based PU is far less documented and was usually achieved by fungal activity (Matsumiya et al., 2010; Álvarez-Barragán et al., 2016).

The biodegradation of synthetic polymers in general is a two-step process. It involves the attack by extracellular enzymes overcoming the macromolecular structure of the polymers and providing monomers and oligomers for the second step, which is the mineralization of the latter inside the cell. The two steps can be carried out by a single species, or more likely by at least two. Regularly, aromatic monomers are released by the activity of extracellular enzymes. During microbial degradation of aromatic compounds typically mono- and dioxygenases are involved in ring hydroxylation and cleavage. The hydroxylation of the aromatic ring results in catecholic compounds (with at least two adjacent hydroxyl groups) reducing the aromatic character of the compound and facilitating the oxygenolytic cleavage of the ring. The latter can be intradiolic (*ortho*-cleavage) or extradiolic (*meta*-cleavage).

Studies that identified the products of PU hydrolysis found the diamines TDA and MDA (Matsumiya et al., 2010; Cregut et al., 2013; Magnin et al., 2019). Both amines have been proposed in the European Chemicals Agency to be identified as “Substances of Very High Concern,” specifically in the category of “Carcinogenic, Mutagenic or toxic to Reproduction” (European Chemicals Agency, 2019). The carcinogenicity of TDA compounds was demonstrated with experimental studies in animals (Baua, 2008). To understand the fate of the diamines released from PU degradation and in order to investigate the monomer and oligomer metabolism in plastics degradation in general, we screened for bacteria capable to degrade both, 2,4-TDA and PU oligomer (Polyurethane diol solution, Sigma-Aldrich). From a site rich in brittle plastic waste, a *Pseudomonas* species was isolated on 2,4-TDA and positively tested for growth on the PU oligomer as the sole source of carbon and energy. Genome sequencing and the screening for potential carbon substrates led to a hypothetic degradation pathway of 2,4-TDA in the isolated *Pseudomonas* strain.

## Materials and Methods

### Growth Conditions

The bacteria were grown in mineral media, as reported before (Hartmans et al., 1989), containing the following compounds (per liter demineralized water): 7 g Na_2_HPO_4_ × 2 H_2_O; 2.8 g KH_2_PO_4_; 0.5 g NaCl; 0.1 g NH_4_Cl; 0.1 g MgSO_4_ × 7 H_2_O; 10 mg FeSO_4_; 5 mg MnSO_4_; 6.4 mg ZnCl_2_; 1 mg CaCl_2_ × 6 H_2_O; 0.6 mg BaCl_2_; 0.36 mg CoSO_4_ × 7 H_2_O; 0.36 mg CuSO_4_ × 5 H_2_O; 6.5 mg H_3_BO_3_; 10 mg EDTA; 146 μl HCl (37%). The nitrogen-deficient mineral media did not contain NH_4_Cl. As sole source of carbon and energy either 4 g/l disodium succinate (Sigma-Aldrich), 2 mM 2,4-TDA (Sigma-Aldrich) or 3 g/l PU oligomer (Sigma-Aldrich, dihydroxy-functional oligomer, aliphatic urethane of proprietary composition) was added. For growth on solid media 3.5% of agar was added. Cells were cultivated in 50 ml shaking cultures at 30°C at 150 rpm. All chemicals used were reagent grade and obtained from commercial sources. Optical density was measured at a wavelength of 560 nm (Perkin Elmer, Lambda 2S). Toluene, benzene, aniline, 2,4-dihydroxytoluene (4-methylresorcinol), methylsuccinate, sodium benzoate, 2-aminobenzoate (anthranilate), phenol, *o*-xylene, catechol, 4-methylcatechol and benzene-1,2,4-triol (hydroxyhydroquinone) were tested if they serve as sole source of carbon and energy for the isolated bacteria in 100, 200, and 300 mg/l concentrations and OD at 560 nm was measured to evaluate growth.

### Bacterial Strain Isolation and Identification

For the isolation of bacteria from soil, three samples from a site rich in brittle plastic waste (Paunsdorf, Leipzig, Germany) were used. 1 g of each sample was dissolved in 9 mL of NaCl 0.9% m/V, diluted 1:10 and stored at 4°C. Afterward, dilution series of 10^–1^, 10^–2^, and 10^–3^ were prepared. 150 μL of the diluted soil solutions were added to agar plates containing mineral medium and different concentrations of 2,4-TDA (2, 5, and 10 mM) as sole carbon and energy source. The plates were stored at 30°C. After 5 days of incubation bacteria were transferred to fresh plates, agar plates without carbon source were used as control. The complete 16S rRNA gene sequence was obtained from the TDA1 genome and used for an alignment with other known Pseudomonas species by making use of the RDP data base (Wang et al., 1997).

### Toxicity Test for 2,4-TDA

In order to test the toxic effect of 2,4-TDA on the isolated strain during growth with the readily metabolizable carbon source disodium succinate (4 g/L), 2,4-TDA was added at different concentrations to exponentially growing cultures as described earlier (Heipieper et al., 1995). The control was a culture growing with succinate as the carbon source without the addition of 2 4-TDA.

### Membrane Lipid Fatty Acid Composition

The membrane fatty acid profile for selected strains was obtained. For the phospholipid fatty acids (PLFA) extraction, bacterial cells were harvested from an overnight culture and then centrifuged for 7 min at 13000 rpm. The pellet was washed with 1.5 mL of 10 mM KNO_3_, centrifuged and PLFA extraction was done as reported before (Bligh and Dyer, 1959), methylation was achieved by addition of 0.6 mL of 20% boron trifluoride in methanol (Morrison and Smith, 1964). The identification and quantification of the fatty acid methyl esters (FAME) was done using gas chromatography with flame ionization detector (GC-FID, Agilent Technologies, 6890N Network GC System, 7683B Series Injector). A CP-Sil 88 column (Varian CP7488) was used as stationary phase and helium as carrier gas. The temperature ramp programmed was: 2 min 40°C isotherm, a gradient increase to 220°C (8°C × min^–1^) and 10 min 220°C isotherm.

### Genome Sequencing of Selected Strain

Genomic DNA was extracted (DNeasy^®^ Blood & Tissue Kit, QIAGEN) according to the manufacture’s protocol for Gram-negative strains. The quantity of extracted DNA was checked by nanodrop followed by the library preparation with the Nextera XT DNA library kit (Illumina, San Diego, CA, United States). The library was checked with an Agilent technology Bioanalyzer 2100. Paired-end libraries were sequenced using Illumina v3 chemistry on a Illumina MiSeq sequencer with a 250-bp paired-end protocol according to the manufacturer’s instructions. The sequencing reads were demultiplexed by MiSeq reporter software (Illumina). The draft genome sequences were assembled using the Velvet assembly program (Zerbino and Birney, 2008). The RAST queue (Aziz et al., 2008) was used to annotate by using *P. putida* KT2440 as reference strain. For the annotation of dioxygenases the AROMADEG data base was used in addition (Duarte et al., 2014). To reveal similarities to known enzymes (mono- and dioxygenases, enzymes involved in aromatics degradation) amino acid sequences of genes present in the genome of TDA1 were compared to UniprotKB database or by using the basic local alignment search tool (BLAST) data base in NCBI as reported before (Altschul et al., 1997). The suggestion of genes possibly involved in the degradation was based on significant amino acid sequence similarities, i.e., a high coverage (at least 80%) and similarity (at least 30%) as well as a low *E* value (1 × 10^–8^ or lower) given by BLAST when compared to the sequences to known and described enzymes. Dioxygenases or enzymes with an aromatic substrate were analyzed mainly by deploying the AROMADEG data base.

## HPLC Measurements

2,4-TDA degradation was monitored by measuring the decrease in concentration. The experiment was performed in triplicates. 50 mL of 2 mM 2,4-TDA media were inoculated with the isolated bacterial strain. 1 mL of the culture was collected and mixed with an equal amount of methanol. A calibration curve for the concentrations between 0.1 mM and 3 mM of 2,4-TDA was prepared. All the samples were centrifuged (7 min, 13000 rpm) at room temperature and filtered through a 0.45 μm polyethersulfone membrane syringe filter (Whatman^TM^-GE Healthcare). 75 μl of the sample was analyzed by high performance liquid chromatography (HPLC; LC- 20AB, Shimadzu). All the samples and standards were measured using a C18 column (LiChroCART^®^ 125-4, RP-18e, 5 μm, Merck KGaA). Isocratic elution of 2,4-TDA was conducted with 39.5% methanol, 59.5% distilled water and 1.0% triethylamine at a flow rate of 0.65 ml min^–1^ (Freedman et al., 1996). The temperature of the column was kept constant at 25°C. Detection was done with a photodiode array detector, using a deuterium lamp as light source, at 278 nm (SPD-M20A, Shimadzu).

## Results

**FIGURE 1:**
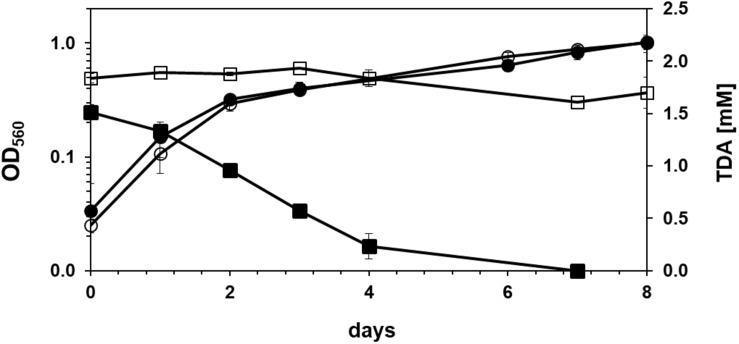
Growth of *Pseudomonas* sp. TDA1 on 2,4-TDA. Circles: Growth of *Pseudomonas* sp. TDA1 on 2 mM 2,4-TDA as sole source of carbon and energy in mineral medium containing an additional nitrogen source (filled circles) and in nitrogen deficient mineral media (empty circles). Squares: Consumption of 2,4-TDA measured via HPLC during the course of cultivation of *Pseudomonas* sp. TDA1 on 1.5 mM 2,4-TDA as sole source of carbon and energy in mineral medium (filled squares) or in a sterile control containing 1.8 mM 2,4-TDA (empty squares) *n* = 3.

**FIGURE 2:**
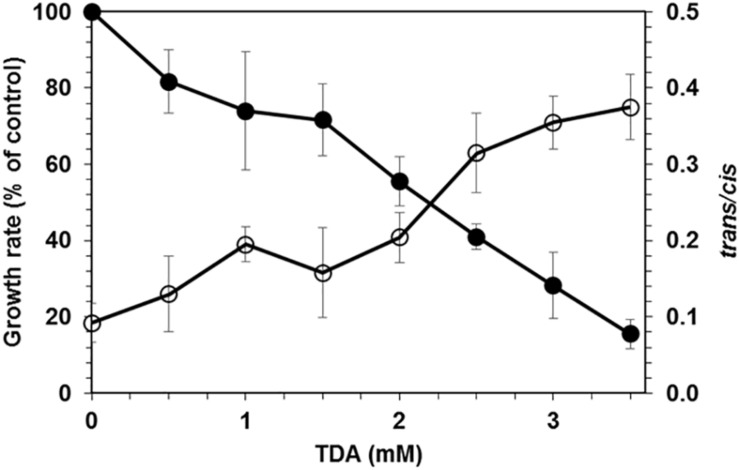
Effect of 2,4-TDA on *P. putida* KT2440. Filled circles: Effect of 2,4-TDA on growth of *P. putida* KT2440. The growth rate after the addition of 2,4-TDA to exponentially growing cells is given relative to a control without 2,4-TDA. Empty circles: Effect of different 2,4-TDA concentrations on the *trans/cis* ratio of unsaturated fatty acids of *P. putida* KT2440.

The screening performed with soil samples taken from a site rich in brittle plastic waste led to the isolation of two bacterial strains that grew on agar plates containing mineral medium with 2,4-TDA as sole carbon and energy source and showed growth in liquid media containing 2 mM 2,4-TDA. Any isolated bacteria that did grow on agar plates without any carbon source were discarded to exclude autotrophic growth on 2,4-TDA agar plates. One strain, named TDA1, was chosen for further investigations. Figure 1 shows the growth of the TDA1 isolate on 2 mM 2,4-TDA as sole carbon and energy source. The growth rate was 0.04 h^–1^ corresponding to a generation time of 14 h^–1^ during exponential growth phase. The degradation of 2,4-TDA was quantified using HPLC. The 2,4-TDA was consumed by the bacterial strain whereas the sterile control only shows a minor decrease in 2,4-TDA concentrations (Figure 1). 2,4-TDA at a concentration of 2 mM was shown to be the optimal concentration, because lower and higher concentrations yielded lower optical densities (data not shown). This was also verified in toxicity tests where 2,4-TDA was added to cells growing exponentially with succinate as carbon and energy source (Figure 2). The growth rate with succinate in the presence of 2 mM 2,4-TDA was reduced by 55% compared to the untreated control whereas higher concentrations caused significantly higher growth inhibition.

Remarkably, strain TDA1 was also able to grow in a nitrogen-deficient mineral media containing only 2,4-TDA as sole carbon and nitrogen source. Optical densities were similar to those obtained with ammonium chloride as nitrogen source (Figure 1). Next to 2,4-TDA also other (aromatic) compounds were tested if they serve as sole source of carbon and energy for the isolated strain. Toluene, benzene, aniline, 2,4-dihydroxytoluene and methylsuccinate did not support growth of the TDA1 strain, whereas benzoate, 2-aminobenzoate (anthranilate), phenol, *o*-xylene, catechol, 4-methylcatechol and benzene-1,2,4-triol served as a growth substrate (Table 1). In addition, the strain grew on an aliphatic oligomeric PU substrate of proprietary composition (PU diol solution, Sigma-Aldrich). Optical densities of about 0.8 were obtained with a concentration of 3 g/l (about 9 mM) of the oligomeric PU as sole carbon and energy source (data not shown).

**TABLE 1:** Growth spectrum for *Pseudomonas* sp. TDA1.

Carbon source	Growth
Toluene	−
Benzene	−
Aniline	−
2,4-Dihydroxytoluene (4-Methylresorcinol)	−
Methylsuccinate	−
Sodium benzoate	+
2-Aminobenzoate (Anthranilate)	+
Phenol	+
*o*-Xylene	+
Catechol	+
4-Methylcatechol	+
Benzene-1,2,4-triol (Hydroxyhydroquinone)	+

*Aromatic substrates that were tested as sole source of carbon and energy for strain TDA1. Plus, growth. Minus, no growth.*

The whole genome sequence has been deposited at DDBJ/ENA/GenBank under the accession WOVH00000000. The version described in this paper is version WOVH01000000. The gene locus tag is GNP06_XXXXX, the corresponding five-digit number is given in the text for each gene mentioned. Using the complete 16S rRNA gene sequence (gene 02555), the strain was identified as *Pseudomonas* sp. strain that shows high similarity with *P. oryzihabitans* and various *P. putida* strains. The strain TDA1 will be referred to as *Pseudomonas* sp. TDA1 in this paper. In addition, the phospholipid fatty acid profile of the strain TDA1 showed the presence of the following fatty acids: C14:0, C16:0, C16:1*trans*, C16:1*cis*, 17*cyclo*, C18:0, C18:1*trans*, C18:1*cis*, and 19*cyclo* (data not shown) comprising more than 95% of the total fatty acids of the strain. The fatty acid composition and pattern of TDA1 was the same as the one of strain *P. putida* KT2440 which was used as a control and benchmark. In addition, the gene for the *cis-trans* isomerase of unsaturated fatty acids (CTI), an important marker gene for the genus *Pseudomonas* (Palleroni, 2015; Eberlein et al., 2018), is present in the TDA1 genome (gene 13840) revealing more than 90% amino acid sequence identity with several *Pseudomonas* CTIs already present in the protein BLAST database (for example: Accession numbers Q8RJN7, A0A059V043, and F8FYU0). Also, the CTI phenotype, regularly given as solvent stress-depending increase in the *trans/cis* ratio of unsaturated fatty acids, was detected in the presence of 2,4-TDA in *P. putida* KT2440 (Figure 2).

Among pathways for degradation of central catecholic intermediates, genes encoding enzymes of the catechol branch of the 3-oxoadipate pathway (catechol 1,2-dioxygenase, muconate cycloisomerase and muconolactone isomerase, genes 25335, 25340, 25345) as well as those encoding the protocatechuate branch (α- and β-subunit of protocatechuate 3,4-dioxygenase, 3-carboxymuconate cycloisomerase and 4-carboxymuconolactone decarboxylase; genes 17435, 17430, 07520, and 07510) and the respective 3-oxoadipate enol-lactone hydrolases (genes 20490 and 07515, respectively) were identified. In addition, genes encoding enzymes for the formation of homogentisate (4-hydroxyphenylpyruvate dioxygenases, genes 05520 and 05730) and a homogentisate 1,2-dioxygenase pathway (genes 17645, 17650, and 17655) as well as a homoprotocatechuate pathway including a LigB type 3,4-dihydroxyphenylacetate 2,3-dioxygenase (gene 05110) were observed. Genes encoding enzymes of the corresponding *meta*-cleavage pathway for homoprotocatechuate were found: 5-carboxymethyl-2-hydroxymuconate semialdehyde dehydrogenase (gene 05115), 5-carboxymethyl-2-hydroxymuconate isomerase (gene 05105), 5-carboxymethyl-2-oxo-hex-3-ene-1,7-dioate decarboxylase (genes 05120 or 05125), and 2-oxo-hepta-3-ene-1,7-dioic acid hydratase (gene 05095). An additional dioxygenase was identified (gene 06545) which according to AROMADEG (Duarte et al., 2014) belongs to a family of extradiol dioxygenases of the vicinal oxygen chelate superfamily of extradiol dioxygenases comprising, among others, enzymes using miscellaneous substrates such as 2,3-dihydroxybenzoate and clustered with proteobacterial extradiol dioxygenases of unknown function (comprising among others YP_046462, an extradiol dioxygenase of *Acinetobacter baylyi* ADP1).

Genes coding for archetype catechol 2,3-dioxygenases such as *xylE, catE*, or *nahA*, extradiol dioxygenases belonging to family I.2 of the vicinal oxygen chelate superfamily, showing a preference for monocyclic substrates and specifically cluster in XXII according to the revised phylogeny of AROMADEG (Eltis and Bolin, 1996; Vaillancourt et al., 2004; Pérez-Pantoja et al., 2009), are not present in the genome of TDA1. Neither ring cleaving dioxygenases involved in aminoaromatic degradation like 5-aminosalicylate 1,2 dioxygenase (Stolz and Knackmuss, 1993), 2-aminophenol 1,6-dioxygenase (Takenaka et al., 1997; Wu et al., 2005) nor hydroxybenzoquinol 1,2-dioxygenase (Travkin et al., 1997; Kitagawa et al., 2004; Wang et al., 2007; Pérez-Pantoja et al., 2009) are encoded in the TDA1 genome.

At least seven genes encoding putative α-subunits of Rieske non-heme iron dioxygenases are present in the genome of TDA1. They were analyzed using AROMADEG (Duarte et al., 2014): it was shown, that genes 26235, 17905, and 06615 are distantly related to enzymes of the phthalate family of Rieske dioxygenases. Gene 26235 probably encodes a vanillate O-demethylase with 76.2% amino acid sequence similarity to P12609 from *Pseudomonas* strain ATCC 19151. The product of gene 06615 shows significant amino acid sequence similarity of 47% with toluene 4-sulfonate monooxygenase TsaM1 (accession P94679) from *Comamonas testosteroni* T-2 (Locher et al., 1991a, b). Among enzymes of documented function, also the product of gene 17905 shows similarity to toluene 4-sulfonate monooxygenase TsaM1, however, only to a low extent of 33%. The gene product of 06600 clearly is a member of the phthalate family of Rieske oxygenases. According to AROMADEG, it belongs to a cluster comprising putative phthalate 4,5-dioxygenase from *Ralstonia eutropha* JMP134 (accession YP298987). Gene 19420 encodes a protein with 73.8% similarity with CntA carnitine monooxygenase (accession D0C9N6) of *Acinetobacter baumannii* ATCC 19606 and thus may be responsible for carnitine transformation to form trimethylamine and malic semialdehyde. The protein encoded by gene 25270 belongs to cluster I of the benzoate family of Rieske dioxygenases (enzymes involved in indole acetic acid degradation and related enzymes). Gene 08315 encodes a benzoate 1,2-dioxygenase (cluster XI, benzoate and 2-chlorobenzoate dioxygenases of the benzoate family of Rieske dioxygenases) with 97.1% identity with BenA of *P. putida* GJ31 (accession AAX47023).

Neither gene clusters encoding proteins involved in the side-chain oxidation of methyl-substituted aromatics, namely the two-component xylene/*p*-cymene monooxygenase, which consist of a hydroxylase related to AlkB alkane hydroxylase and a reductase (Worsey and Williams, 1975; Eaton, 1996) were observed in the genome, nor are multicomponent soluble diiron benzene/toluene or phenol/methylphenol monooxygenases encoded. However, five genes coding for flavin depending monooxygenases were detected (genes 05080, 17225, 06905, 06505, 06585). Gene products of 05080 and 06585 show high amino acid sequence similarity to 4-hydroxyphenylacetate 3-hydroxylase from *Acinetobacter baumannii* (accession Q6Q272) of 72.1% and 72.6%, respectively (Thotsaporn et al., 2004). The product of gene 17225 exhibits high sequence identity to documented 4-hydroxybenzoate 3-monooxygenases such as the enzymes P00438 from *P. fluorescens* (74.9%) or P20586 from *P. aeruginosa* (74.6%). In contrast to that, the function of flavin monooxygenases 06505 and 06905 remains unknown.

The release of nitrogen from aromatic amines can occur before ring cleavage in form of ammonia (Aoki et al., 1983; Chang et al., 2003; Takenaka et al., 2003), but also after ring opening (Takenaka et al., 2000). The latter is done by 2-aminomuconate deaminase during 2-aminophenol degradation by *Pseudomonas* sp. AP-3. This enzyme belongs to the YjgF/YER057c/UK114 family (also known as the Rid family). Five members of this family were observed to be encoded in the genome of the strain TDA1 (genes 01225, 03255, 14860, 17920, 05035). For two of these gene products significant similarities to 2-aminomuconate deaminase of *Pseudomonas* sp. AP–3 (accession Q9KWS2) could be documented: 36% for the gene product of 14860 and 32% for the gene product of 05035.

## Discussion

A bacterial strain capable of degrading both, an oligomeric PU and a PU building block was obtained from soil samples. According to our knowledge, this is the first report on the isolation of a bacterial pure culture for the polyurethane precursor 2,4-TDA. A powerful metabolic potential of the strain is given because of the ability to use both as sole source of carbon and energy, a monomer and an oligomer of PU. 2,4-TDA was used not only as the carbon and but also as a nitrogen source. That concentrations higher than 2 mM 2,4-TDA did not increase the optical densities further, might be due to toxic effect. Also for *P. putida* KT2440 it was shown, that concentrations above 2 mM 2,4-TDA diminished growth. The isolate was identified as *Pseudomonas* sp. strain by 16S rRNA gene sequence analysis and by comparing the fatty acid profile to the one of *P. putida* KT2440. The isolation of a *Pseudomonas* strain from the same oligomeric PU material was reported before (Mukherjee et al., 2011). Moreover, microbial attack on polyurethanes by species of the genus *Pseudomonas* was documented earlier (Howard and Blake, 1998; Howard, 2002; Gautam et al., 2007; Peng et al., 2014; Hung et al., 2016). The fact that PU polymers or components do not only meet the carbon but also the nitrogen demand was confirmed in this study. Earlier reports also had shown that polyisocyanates may serve as nitrogen source (Darby and Kaplan, 1968; Crabbe et al., 1994; Nakajima-Kambe et al., 1995; Kloss et al., 2009).

**FIGURE 3:**
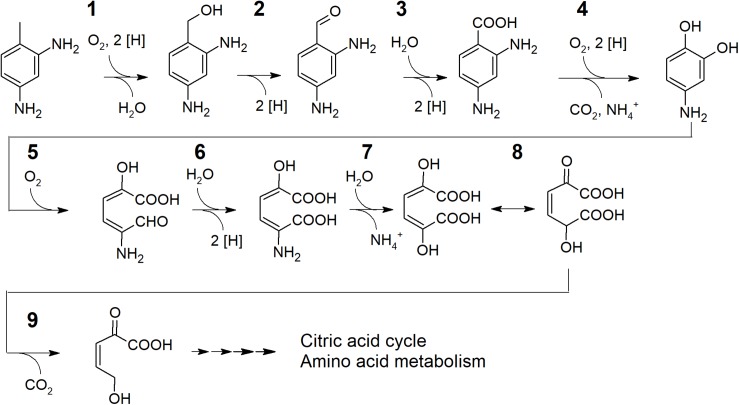
Proposed degradation pathway including extradiol cleavage of 4-aminocatechol for 2,4-TDA in the putative *Pseudomonas* sp. TDA1. **1**: Toluene 4-sulfonate monooxygenase (candidate gene GNP06_06615) with toluene-4-sulfonate monooxygenase reductase (candidate gene GNP06_06620) and subsequent alcohol dehydrogenase **(2)** and aldehyde dehydrogenase **(3)** activity (encoded elsewhere in the genome). **4**: Benzoate 1,2-dioxygenase with anthranilate dioxygenase activity (candidate genes for α- and β-subunit as well as a ferredoxin reductase component GNP06_08305, GNP06_08310 and GNP06_08315). **5**: Extradiol 2,3-dioxygenase (candidate gene GNP06_06545 or GNP06_05110). **6**, **8** and **9**: Enzymes for the homoprotocatechuate *meta*-cleavage pathway (candidate genes GNP06_05115, GNP06_05105 and GNP06_05120/25). **7**: 4-amino-2-hydroxymuconate deaminase (candidate genes GNP06_14860 or GNP06_05035).

Considering the genomic potential and the substrate spectrum a degradation pathway for 2,4-TDA with candidate genes encoding the enzymes involved can be suggested (Figure 3). Although also a monooxygenation of an aromatic ring lacking hydroxyl groups has been reported in the case of styrene (Beltrametti et al., 1997), an initiation of the degradation of not yet activated aromatics by flavin monooxygenases is rather unlikely (Van Berkel et al., 2006). In contrast to that, hydroxylation of substituents at the aromatic ring, like the methyl group of toluene, is common (Assinder and Williams, 1990). However, strain TDA1 does not grow on toluene (Table 1) and the only putative methyl group oxidizing enzymes encoded are those with similarity to toluene 4-sulfonate monooxygenase TsaM1 (accession P94679) from *Comamonas testosteroni* T-2 (Locher et al., 1991a, b). Therefore, it can be assumed that the methyl group is hydroxylated to a primary alcohol (candidate gene 06615) with the help of an electron transferring unit. For the latter, a gene encoding for a protein sharing 48.1% sequence similarity with toluene-4-sulfonate monooxygenase reductase subunit TsaB1 (accession P94680) in *Comamonas testosteroni* is located adjacent to 06615. Obviously, the methyl oxidizing enzyme present needs a substituent in *para* position on the aromatic ring to function as the strain cannot grow on toluene. The following steps yielding 2,4-diaminobenzoate (4-aminoanthranilate) would be catalyzed by an alcohol dehydrogenase and subsequently by an aldehyde dehydrogenase encoded elsewhere in the genome. Strain TDA1 uses anthranilate as the sole source of carbon and energy which typically is catalyzed by an anthranilate 1,2-dioxygenase (Cain, 1968; Eby et al., 2001; Schühle et al., 2001; Chang et al., 2003; Liu et al., 2010; Costaglioli et al., 2012; Kim et al., 2015). No such enzyme is encoded in the genome of TDA1. However, some benzoate dioxygenases are reported to transform anthranilate to catechol (Yamaguchi and Fujisawa, 1980; Haddad et al., 2001) and a gene cluster encoding a benzoate 1,2-dioxygenase α- and β-subunit as well as a ferredoxin reductase component (genes 08305, 08310, 08315) is conserved in the genome. It is therefore conceivable that 2,4-diaminobenzoate is transformed by benzoate 1,2-dioxygenase yielding 4-aminocatechol as central intermediate.

Studies showed that aromatic compounds with electron-donating substituents, such as amino groups, are preferably degraded via the *meta*-cleavage pathway (Ribbons, 1965; Seidman et al., 1969; Bugg and Ramaswamy, 2008; Shukla et al., 2016). It can therefore be speculated that the putative intermediate 4-aminocatechol is transformed by an extradiol dioxygenase; and a respective extradiol dioxygenase of the vicinal chelate superfamily is actually encoded in the genome (candidate gene 06545). A second extradiol dioxygenase, a homoprotocatechuate 2,3-dioxygenase of the LigB superfamily (Roper and Cooper, 1990), is encoded by gene 05110 located within a gene cluster encoding enzymes for the further metabolism of the homoprotocatechuate ring-cleavage product via the *meta*-cleavage pathway. Several publications state that homoprotocatechuate 2,3-dioxygenase is promiscuous and may accept 4-nitrocatechol as a substrate (Groce et al., 2004; Henderson et al., 2012; Kovaleva and Lipscomb, 2012; Mbughuni et al., 2012). If the 06545 extradiol dioxygenase or a promiscuous homoprotocatechuate dioxygenase is involved in 2,4-TDA degradation by strain TDA1 remains to be elucidated. Further degradation of the putative ring-cleavage product 4-amino-2-hydroxymuconate semialdehyde may then be performed by homoprotocatechuate *meta*-cleavage pathway enzymes with 5-carboxymethyl-2-hydroxymuconic semialdehyde dehydrogenase encoded by gene 05115 forming 4-amino-2-hydroxymuconate. As the next step, the formed 4-amino-2-hydroxymuconate could be deaminated by an aminomuconate deaminase (candidate genes 14860 or 05035) similar to the deamination after ring cleavage in the degradation pathway of aminophenol in *Pseudomonas* sp. AP-3 (Takenaka et al., 2000) or in nitrobenzene degradation in *Pseudomonas pseudoalcaligenes* JS4 (He and Spain, 1997). For the latter, the enzyme 2-aminomuconate deaminase does not depend on cofactors and deamination of its substrate even happens spontaneously in acidic environments (Ichiyama et al., 1965). In the metabolization of 4-amino-3-hydroxybenzoic acid in *Bordetella* sp. 10d the amino group is cleaved off already from the muconic semialdehyde intermediate by a 2-amino-5-carboxymuconicsemialdehyde deaminase (Orii et al., 2006). The resulting intermediate 2,5-dihydroxy-muconate probably undergoes tautomerization (gene 05105) and could be further subjected to a decarboxylation step (gene 05120 or 05125). Following the *meta-*cleavage pathway, a hydroxylation would take place after the decarboxylation and the corresponding hydratase is also present in the genome of TDA1 (gene 05095). However, how exactly the degradation pathway is continued to lead to central metabolites of the citric acid cycle or amino acid metabolism needs to be elucidated in further studies.

To sum up, a preliminary degradation pathway of 2,4-TDA is proposed. In the peripheral pathway 4-aminocatechol is formed after oxidation of the methyl group of diaminotoluene and subsequent dioxygenation with concomitant decarboxylation and deamination. Ring cleavage of 4-aminocatechol in TDA1 would be possible in an extradiol manner (candidate gene 06545) and further employment of the homoprotocatechuate *meta*-pathway (genes 05115, 05105, 05120/25) with the second deamination potentially taking place after the formation of 5-amino 2-hydroxymuconate (candidate genes 14860 or 05035).

The majority of the enzymes involved in the proposed pathway must be promiscuous regarding their substrate specificity, i.e., they need to accept especially amino substituted analogs. Due to the low steric hindrance of an additional amino group substrate promiscuity might be favored. Enzymes involved in aromatics degradation exhibiting significant activity with substituted substrate analogs were reported before (Pascal and Huang, 1986; Smith et al., 1990; He and Spain, 1997; Eby et al., 2001; Chang et al., 2003; Guzik et al., 2011). However, the proposed degradation pathway of 2,4-TDA in the putative *Pseudomonas* strain TDA1 needs further confirmation via proteomic, transcriptomic analysis or *in vitro* assays with potential intermediates of the proposed pathway. Identifying the key enzymes for the degradation of both, 2,4-TDA as putative degradation product as well as precursor of PUs (Matsumiya et al., 2010; Magnin et al., 2019) and for the oligomeric PU could help to equip well known and biotechnological used lab strains like *P. putida* KT2440 for monomer degradation in two-step biorecycling processes.

## Data Availability Statement

The whole genome sequence has been deposited at DDBJ/ENA/GenBank under the accession WOVH00000000. The version described in this article is version WOVH01000000.

## Author Contributions

MC, CE, UK, and HH conceived and designed the experiments. MC, AC, TS, and AA-K performed the experiments. DT performed the genome sequencing and annotation. DP, UK, CE, and HH analyzed the data. HH and CE contributed reagents, materials, and analysis tools. MC, CE, DP, and HH wrote the manuscript.

## Conflict of Interest

The authors declare that the research was conducted in the absence of any commercial or financial relationships that could be construed as a potential conflict of interest.

## References

[B1] Altschul S. F. Madden T. L. Schaffer A. A. Zhang J. H. Zhang Z. Miller W. (1997). Gapped BLAST and PSI-BLAST: a new generation of protein database search programs. *Nucleic Acids Res.* 25 3389–3402. 9254694 10.1093/nar/25.17.3389 PMC146917

[B2] Álvarez-Barragán J. Domínguez-Malfavon L. Vargas-Suárez M. González-Hernández R. Aguilar-Osorio G. Loza-Tavera H. (2016). Biodegradative activities of selected environmental fungi on a polyester polyurethane varnish and polyether polyurethane foams. *Appl. Environ. Microbiol.* 82 5225–5235. 10.1128/AEM.01344-16 27316963 PMC4988181

[B3] Aoki K. Shinke R. Nishira H. (1983). Microbial-metabolism of aromatic-amines.3. Metabolism of Aniline by Rhodococcus-Erythropolis an-13. *Agricult. Biol. Chem.* 47 1611–1616.

[B4] Assinder S. J. Williams P. A. (1990). The tol plasmids - determinants of the catabolism of toluene and the xylenes. *Adv. Microb. Physiol.* 31 1–69. 2264522 10.1016/s0065-2911(08)60119-8

[B5] Aziz R. K. Bartels D. Best A. A. Dejongh M. Disz T. Edwards R. A. (2008). The RAST server: rapid annotations using subsystems technology. *Bmc Genomics* 9:75. 10.1186/1471-2164-9-75 18261238 PMC2265698

[B6] Baua T. (2008). *4-Diamine: Summary Risk Assessment Report.* Avaliable at: https://echa.europa.eu/documents/10162/a306907a-8401-4a75-8a84-88b9f225d5cf (accessed October 2019).

[B7] Beltrametti F. Marconi A. M. Bestetti G. Colombo C. Galli E. Ruzzi M. (1997). Sequencing and functional analysis of styrene catabolism genes from *Pseudomonas fluorescens* ST. *Appl. Environ. Microbiol.* 63 2232–2239. 9172343 10.1128/aem.63.6.2232-2239.1997 PMC168516

[B8] Bligh E. G. Dyer W. J. (1959). A rapid method of total lipid extraction and purification. *Can. J. Biochem. Physiol.* 37 911–917. 13671378 10.1139/o59-099

[B9] Bugg T. D. Ramaswamy S. (2008). Non-heme iron-dependent dioxygenases: unravelling catalytic mechanisms for complex enzymatic oxidations. *Curr. Opin. Chem. Biol.* 12 134–140. 10.1016/j.cbpa.2007.12.007 18249197

[B10] Cain R. B. (1968). Anthranilic acid metabolism by microorganisms. formation of 5-hydroxyanthranilate as an intermediate in anthranilate metabolism by *Nocardia Opaca*. *Antonie Van Leeuwenhoek* 34 417–432. 5304014

[B11] Chang H. K. Mohseni P. Zylstra G. J. (2003). Characterization and regulation of the genes for a novel anthranilate 1,2-dioxygenase from *Burkholderia cepacia* DBO1. *J. Bacteriol.* 185 5871–5881. 13129960 10.1128/JB.185.19.5871-5881.2003 PMC193950

[B12] Costaglioli P. Barthe C. Claverol S. Brozel V. S. Perrot M. Crouzet M. (2012). Evidence for the involvement of the anthranilate degradation pathway in *Pseudomonas aeruginosa* biofilm formation. *Microbiol. Open* 1 326–339. 10.1002/mbo3.33 23170231 PMC3496976

[B13] Crabbe J. R. Campbell J. R. Thompson L. Walz S. L. Schultz W. W. (1994). Biodegradation of a Colloidal Ester-Based Polyurethane by Soil Fungi. *Int. Biodeter Biodegr.* 33 103–113.

[B14] Cregut M. Bedas M. Durand M. J. Thouand G. (2013). New insights into polyurethane biodegradation and realistic prospects for the development of a sustainable waste recycling process. *Biotechnol. Adv.* 31 1634–1647. 10.1016/j.biotechadv.2013.08.011 23978675

[B15] Darby R. T. Kaplan A. M. (1968). Fungal susceptibility of polyurethanes. *Appl. Microbiol.* 16 900–910. 16349806 10.1128/am.16.6.900-905.1968 PMC547551

[B16] Duarte M. Jauregui R. Vilchez-Vargas R. Junca H. Pieper D. H. (2014). AromaDeg, a novel database for phylogenomics of aerobic bacterial degradation of aromatics. *Database* 2014:bau118. 10.1093/database/bau118 25468931 PMC4250580

[B17] Eaton R. W. (1996). p-Cumate catabolic pathway in Pseudomonas putida Fl: cloning and characterization of DNA carrying the cmt operon. *J. Bacteriol.* 178 1351–1362. 10.1128/jb.178.5.1351-1362.1996 8631713 PMC177810

[B18] Eberlein C. Baumgarten T. Starke S. Heipieper H. J. (2018). Immediate response mechanisms of Gram-negative solvent-tolerant bacteria to cope with environmental stress: cis-trans isomerization of unsaturated fatty acids and outer membrane vesicle secretion. *Appl. Microbiol. Biotechnol.* 102 2583–2593. 10.1007/s00253-018-8832-9 29450619 PMC5847196

[B19] Eby D. M. Beharry Z. M. Coulter E. D. Kurtz D. M. Jr. Neidle E. L. (2001). Characterization and evolution of anthranilate 1,2-dioxygenase from *Acinetobacter* sp. strain ADP1. *J. Bacteriol.* 183 109–118. 11114907 10.1128/JB.183-1.109-118.2001 PMC94856

[B20] Eltis L. D. Bolin J. T. (1996). Evolutionary relationships among extradiol dioxygenases. *J. Bacteriol.* 178 5930–5937. 8830689 10.1128/jb.178.20.5930-5937.1996 PMC178449

[B21] European Chemicals Agency (2019). *European Chemicals Agency.* Avaliable at: https://echa.europa.eu/home (accessed October 2019).

[B22] Vaillancourt F. H. Bolin J. T. Eltis L. D. (2004). “Ring-cleavage dioxygenases,” in *Pseudomonas*, ed. Ramos J. (New York, NY: Kluwer Academic/Plenum Publishers), 359–395.

[B23] Freedman D. L. Shanley R. S. Scholze R. J. (1996). Aerobic biodegradation of 2,4-dinitrotoluene, aminonitrotoluene isomers, and 2,4-diaminotoluene. *J. Hazard Mater.* 49 1–14.

[B24] Gautam R. Bassi A. S. Yanful E. K. Cullen E. (2007). Biodegradation of automotive waste polyester polyurethane foam using *Pseudomonas chlororaphis* ATCC55729. *Int. Biodeter. Biodegr,.* 60 245–249.

[B25] Groce S. L. Miller-Rodeberg M. A. Lipscomb J. D. (2004). Single-turnover kinetics of homoprotocatechuate 2,3-dioxygenase. *Biochemistry* 43 15141–15153. 15568806 10.1021/bi048690x

[B26] Guzik U. Greń I. Hupert-Kocurek K. Wojcieszyńska D. (2011). Catechol 1,2-dioxygenase from the new aromatic compounds – Degrading *Pseudomonas putida* strain N6. *Int. Biodeterioration Biodegrad.* 65 504–512. 10.1016/j.ibiod.2011.02.001

[B27] Haddad S. Eby D. M. Neidle E. L. (2001). Cloning and expression of the benzoate dioxygenase genes from *Rhodococcus* sp. strain 19070. *Appl. Environ. Microbiol.* 67 2507–2514. 11375157 10.1128/AEM.67.6.2507-2514.2001 PMC92901

[B28] Hartmans S. Smits J. P. Van Der Werf MJ. Volkering F. De Bont J. A. M. (1989). Metabolism of styrene oxide and 2-phenylethanol in the styrene-degrading *Xanthobacter* strain 124X. *Appl. Environ. Microbiol.* 55 2850–2855. 16348047 10.1128/aem.55.11.2850-2855.1989 PMC203180

[B29] He Z. G. Spain J. C. (1997). Studies of the catabolic pathway of degradation of nitrobenzene by *Pseudomonas pseudoalcaligens* JS45: removal of the amino group from 2-aminomuconic semialdehyde. *Appl. Environ. Microbiol.* 63 4839–4843. 9471964 10.1128/aem.63.12.4839-4843.1997 PMC168809

[B30] Heipieper H. J. Loffeld B. Keweloh H. De Bont J. A. M. (1995). The *cis/trans* isomerisation of unsaturated fatty acids in *Pseudomonas putida* S12: an indicator for environmental stress due to organic compounds. *Chemosphere* 30 1041–1051.

[B31] Henderson K. L. Le V. H. Lewis E. A. Emerson J. P. (2012). Exploring substrate binding in homoprotocatechuate 2,3-dioxygenase using isothermal titration calorimetry. *J. Biol. Inorg. Chem.* 17 991–994. 10.1007/s00775-012-0929-5 22915062

[B32] Howard G. T. (2002). Biodegradation of polyurethane: a review. *Int. Biodeter. Biodegr.* 49 245–252.

[B33] Howard G. T. Blake R. C. (1998). Growth of *Pseudomonas fluorescens* on a polyester-polyurethane and the purification and characterization of a polyurethanase-protease enzyme. *Int. Biodeter. Biodegr.* 42 213–220.

[B34] Hung C. S. Zingarelli S. Nadeau L. J. Biffinger J. C. Drake C. A. Crouch A. L. (2016). Carbon catabolite repression and impranil polyurethane degradation in *Pseudomonas protegens* Strain Pf-5. *Appl. Environ. Microbiol.* 82 6080–6090. 27496773 10.1128/AEM.01448-16 PMC5068165

[B35] Ichiyama A. Nakamura S. Kawai H. Honjo T. Nishizuka Y. Hayaishi O. (1965). Studies on the metabolism of the benzene ring of tryptophan in mammalian tissues. ii. enzymic formation of alpha-aminomuconic acid from 3-hydroxyanthranilic acid. *J. Biol. Chem.* 240 740–749. 14275130

[B36] Kim D. Yoo M. Kim E. Hong S. G. (2015). Anthranilate degradation by a cold-adapted *Pseudomonas* sp. *J. Basic Microbiol.* 55 354–362. 10.1002/jobm.201300079 23720227

[B37] Kitagawa W. Kimura N. Kamagata Y. (2004). A novel p-nitrophenol degradation gene cluster from a gram-positive bacterium. Rhodococcus opacus SAO101. *J. Bacteriol.* 186 4894–4902. 15262926 10.1128/JB.186.15.4894-4902.2004 PMC451640

[B38] Kloss J. R. Pedrozo T. H. Follmann H. D. Peralta-Zamora P. Dionísio J. A. Akcelrud L. (2009). Application of the principal component analysis method in the biodegradation polyurethanes evaluation. *Mater. Sci. Eng. C* 29 470–473.

[B39] Kovaleva E. G. Lipscomb J. D. (2012). Structural basis for the role of tyrosine 257 of homoprotocatechuate 2,3-dioxygenase in substrate and oxygen activation. *Biochemistry* 51 8755–8763. 10.1021/bi301115c 23066739 PMC3494287

[B40] Krasowska K. Janik H. Gradys A. Rutkowska M. (2012). Degradation of polyurethanes in compost under natural conditions. *J. Appl. Polym. Sci.* 125 4252–4260.

[B41] Liu X. Dong Y. Li X. Ren Y. Li Y. Wang W. (2010). Characterization of the anthranilate degradation pathway in *Geobacillus thermodenitrificans* NG80-2. *Microbiology* 156 589–595. 10.1099/mic.0.031880-0 19942660

[B42] Locher H. H. Leisinger T. Cook A. M. (1991a). 4-Toluene sulfonate methyl-monooxygenase from *Comamonas testosteroni* T-2: purification and some properties of the oxygenase component. *J. Bacteriol.* 173 3741–3748. 2050632 10.1128/jb.173.12.3741-3748.1991 PMC208003

[B43] Locher H. H. Malli C. Hooper S. W. Vorherr T. Leisinger T. Cook A. M. (1991b). Degradation of p-toluic acid (*p*-toluenecarboxylic acid) and *p*-toluenesulphonic acid via oxygenation of the methyl sidechain is initiated by the same set of enzymes in *Comamonas testostemni* T-2. *J. Gen. Microbio.* 137 221–2208.

[B44] Magnin A. Pollet E. Perrin R. Ullmann C. Persillon C. Phalip V. (2019). Enzymatic recycling of thermoplastic polyurethanes: synergistic effect of an esterase and an amidase and recovery of building blocks. *Waste Manag.* 85 141–150. 10.1016/j.wasman.2018.12.024 30803567

[B45] Marchant R. E. Zhao Q. Anderson J. M. Hiltner A. (1987). Degradation of a Poly(Ether Urethane Urea) Elastomer - Infrared and Xps Studies. *Polymer* 28 2032–2039.

[B46] Matsumiya Y. Murata N. Tanabe E. Kubota K. Kubo M. (2010). Isolation and characterization of an ether-type polyurethane-degrading micro-organism and analysis of degradation mechanism by *Alternaria* sp. *J. Appl. Microbiol.* 108 1946–1953. 10.1111/j.1365-2672.2009.04600.x 19912428

[B47] Mbughuni M. M. Meier K. K. Munck E. Lipscomb J. D. (2012). Substrate-mediated oxygen activation by homoprotocatechuate 2,3-Dioxygenase: intermediates formed by a tyrosine 257 variant. *Biochemistry* 51 8743–8754. 10.1021/bi301114x 23066705 PMC3513391

[B48] Morrison W. R. Smith L. M. (1964). Preparation of fatty acid methyl esters and dimethylacetals from lipids with boron fluoride–methanol. *J. Lipid Res.* 5 600–608. 14221106

[B49] Mukherjee K. Tribedi P. Chowdhury A. Ray T. Joardar A. Giri S.. (2011). Isolation of a *Pseudomonas aeruginosa* strain from soil that can degrade polyurethane diol. *Biodegradation* 22 377–388. 10.1007/s10532-010-9409-1 20803164

[B50] Nakajima-Kambe T. Onuma F. Kimpara N. Nakahara T. (1995). Isolation and characterization of a bacterium which utilizes polyester polyurethane as a sole carbon and nitrogen source. *FEMS Microbiol. Lett.* 129 39–42. 7781989 10.1016/0378-1097(95)00131-N

[B51] Orii C. Takenaka S. Murakami S. Aoki K. (2006). Metabolism of 4-amino-3-hydroxybenzoic acid by *Bordetella* sp. strain 10d: a different modified meta-cleavage pathway for 2-aminophenols. *Biosci. Biotechnol. Biochem.* 70 2653–2661. 17090920 10.1271/bbb.60264

[B52] Palleroni N. J. (2015). “Pseudomonas,” in *Bergey’s Manual of Systematics of Archaea and Bacteria*, eds Whitman W. B. Rainey F. Kämpfer P. Trujillo M. Chun J. De Vos P. Hedlund B. Dedysh S. (Hoboken, NJ: John Wiley & Sons, Inc), 1–105.

[B53] Pascal R. A. Jr. Huang D. S. (1986). Reactions of 3-ethylcatechol and 3-(methylthio)Catechol with catechol dioxygenases. *Arch. Biochem. Biophys.* 248 130–137. 10.1016/0003-9861(86)90409-1 3015028

[B54] Peng Y. H. Shih Y. H. Lai Y. C. Liu Y. Z. Liu Y. T. Lin N. C. (2014). Degradation of polyurethane by bacterium isolated from soil and assessment of polyurethanolytic activity of a *Pseudomonas putida* strain. *Environ. Sci. Pollut. Res. Int.* 21 9529–9537. 10.1007/s11356-014-2647-8 24633845

[B55] Pérez-Pantoja D. Donoso R. Junca H. González B. Pieper D. H. (2009). “Phylogenomics of aerobic bacterial degradation of aromatics,” in *Handbook of Hydrocarbon and Lipid Microbiology*, ed. Timmis K. N. (Berlin: Springer), 1355–1397.

[B56] Plasticseurope (2016). “Plastics – the facts 2016,” in . *An Analysis of European Plastics Production, Demand and Waste Data*, Brussels.

[B57] Ribbons D. W. (1965). Microbiological degradation of aromatic compounds. *Annu. Rep. Prog. Chem.* 62:445.

[B58] Roper D. I. Cooper R. A. (1990). Subcloning and nucleotide sequence of the 3,4-dihydroxyphenylacetate (homoprotocatechuate) 2,3-dioxygenase gene from *Escherichia coli* C. *FEBS Lett.* 275 53–57. 2261999 10.1016/0014-5793(90)81437-s

[B59] Russell J. R. Huang J. Anand P. Kucera K. Sandoval A. G. Dantzler K. W. (2011). Biodegradation of polyester polyurethane by endophytic fungi. *Appl. Environ. Microbiol.* 77 6076–6084. 10.1128/AEM.00521-11 21764951 PMC3165411

[B60] Schühle K. Jahn M. Ghisla S. Fuchs G. (2001). Two similar gene clusters coding for enzymes of a new type of aerobic 2-aminobenzoate (anthranilate) metabolism in the bacterium *Azoarcus evansii*. *J. Bacteriol.* 183 5268–5278. 11514509 10.1128/JB.183.18.5268-5278.2001 PMC95408

[B61] Seidman M. M. Toms A. Wood J. M. (1969). Influence of side-chain substituents on position of cleavage of benzene ring by *Pseudomonas Fluorescens*. *J. Bacteriol.* 97 1192–1197. 5776526 10.1128/jb.97.3.1192-1197.1969 PMC249834

[B62] Shah Z. Krumholz L. Aktas D. F. Hasan F. Khattak M. Shah A. A. (2013). Degradation of polyester polyurethane by a newly isolated soil bacterium. *Bacillus subtilis* strain MZA-75. *Biodeg* 24 865–877. 10.1007/s10532-013-9634-5 23536219

[B63] Shukla A. Singh B. Singh Cameotra S. Kahlon S. R. (2016). *Pseudomonas Oyxgenases: Nature and Function.* Switzerland.Berlin: Springer.

[B64] Smith M. R. Ratledge C. Crook S. (1990). Properties of cyanogen bromide-activated, Agarose-immobilized catechol 1,2-dioxygenase from freeze-dried extracts of *Nocardia* Sp. NCIB 10503. *Enzyme Microbial. Technol.* 12 945–949. 10.1016/0141-0229(90)90114-6

[B65] Stolz A. Knackmuss H. J. (1993). Bacterial Metabolism of 5-aminosalicylic acid - enzymatic conversion to L-Malate. Pyruvate and Ammonia. *J. Gen. Microbiol.* 139 1019–1025. 8336104 10.1099/00221287-139-5-1019

[B66] Takenaka S. Murakami S. Kim Y. J. Aoki K. (2000). Complete nucleotide sequence and functional analysis of the genes for 2-aminophenol metabolism from *Pseudomonas* sp.AP-3. *Arch. Microbiol.* 174 265–272. 11081795 10.1007/s002030000203

[B67] Takenaka S. Murakami S. Shinke R. Hatakeyama K. Yukawa H. Aoki K. (1997). Novel genes encoding 2-aminophenol 1,6-dioxygenase from *Pseudomonas* species AP-3 growing on 2-aminophenol and catalytic properties of the purified enzyme. *J. Biol. Chem.* 272 14727–14732. 9169437 10.1074/jbc.272.23.14727

[B68] Takenaka S. Okugawa S. Kadowaki M. Murakami S. Aoki K. (2003). The metabolic pathway of 4-aminophenol in *Burkholderia* sp strain AK-5 differs from that of aniline and aniline with C-4 substituents. *Appl. Environ. Microbiol.* 69 5410–5413. 12957929 10.1128/AEM.69.9.5410-5413.2003 PMC194951

[B69] Thotsaporn K. Sucharitakul J. Wongratana J. Suadee C. Chaiyen P. (2004). Cloning and expression of p-hydroxyphenylacetate 3-hydroxylase from *Acinetobacter baumannii*: evidence of the divergence of enzymes in the class of two-protein component aromatic hydroxylases. *Biochim. Biophys. Acta* 1680 60–66. 15451173 10.1016/j.bbaexp.2004.08.003

[B70] Travkin V. M. Jadan A. P. Briganti F. Scozzafava A. Golovleva L. A. (1997). Characterization of an intradiol dioxygenase involved in the biodegradation of the chlorophenoxy herbicides 2,4-D and 2,4,5-T. *Febs Lett.* 407 69–72. 9141483 10.1016/s0014-5793(97)00297-4

[B71] Van Berkel W. J. Kamerbeek N. M. Fraaije M. W. (2006). Flavoprotein monooxygenases, a diverse class of oxidative biocatalysts. *J. Biotechnol.* 124 670–689. 16712999 10.1016/j.jbiotec.2006.03.044

[B72] Wang G. B. Labow R. S. Santerre J. P. (1997). Biodegradation of a poly(ester)urea-urethane by cholesterol esterase: Isolation and identification of principal biodegradation products. *J. Biomed. Mater. Res.* 36 407–417. 9260112 10.1002/(sici)1097-4636(19970905)36:3<407::aid-jbm16>3.0.co;2-a

[B73] Wang Q. Garrity G. M. Tiedje J. M. Cole J. R. (2007). Naive Bayesian classifier for rapid assignment of rRNA sequences into the new bacterial taxonomy. *Appl. Environ. Microbiol.* 73 5261–5267. 17586664 10.1128/AEM.00062-07 PMC1950982

[B74] Worsey M. J. Williams P. A. (1975). Metabolism of toluene and xylenes by Pseudomonas putida (arvilla) mt-2: evidence for a new function of the TOL plasmid. *J. Bacteriol.* 124 7–13. 10.1128/JB.124.1.7-13.1975 1176436 PMC235858

[B75] Wu J. F. Sun C. W. Jiang C. Y. Liu Z. P. Liu S. J. (2005). A novel 2-aminophenol 1,6-dioxygenase involved in the degradation of p-chloronitrobenzene by *Comamonas strain* CNB-1: purification, properties, genetic cloning and expression in *Escherichia coli*. *Arch. Microbiol.* 183 1–8. 15580337 10.1007/s00203-004-0738-5

[B76] Yamaguchi M. Fujisawa H. (1980). Purification and characterization of an oxygenase component in benzoate 1,2-dioxygenase system from *Pseudomonas arvilla* C-1. *J. Biol. Chem.* 255 5058–5063. 7372624

[B77] Zerbino D. R. Birney E. (2008). Velvet: algorithms for de novo short read assembly using de Bruijn graphs. *Genome Res.* 18 821–829. 10.1101/gr.074492.107 18349386 PMC2336801

